# A fast and easy strategy for protein purification using “teabags”

**DOI:** 10.1038/srep28887

**Published:** 2016-06-30

**Authors:** M. Castaldo, L. Barlind, F. Mauritzson, P. T. Wan, H. J. Snijder

**Affiliations:** 1 Discovery Sciences, Innovative Medicines and Early Development Biotech Unit, AstraZeneca, Pepparedsleden 1, Mölndal, 431 83, Sweden

## Abstract

Protein purification often involves affinity capture of proteins on stationary resin, alternatively proteins are captured on free flowing resin for subsequent separation from bulk fluid. Both methods require labour and time intensive separation of particulate matter from fluid. We present a method where affinity resin is contained within porous-walled containers, supporting clarification, product recovery, and concentration in a single step with minimal hands-on processing time, without significant investments in equipment.

Protein production starts with the generation of feedstock or biomass containing the protein of interest. Recombinant protein expression has greatly facilitated protein production by increasing the relative fraction of the protein of interest in the feedstock. Subsequent, filtration, fractionation and enrichment steps are utilized prior to chromatographic purification of the target protein to homogeneity. These initial steps almost invariably contain centrifugation steps to separate particulate matter, which would otherwise interfere with down-stream purification and block packed columns. These steps are time consuming and labour intensive and limit overall throughput. We present here an alternative in which affinity resin is confined within a porous walled container that simultaneously allows fluid exchange but excludes cells, debris and particulates in the feedstock.

## Results and Discussion

**Figure 1:**
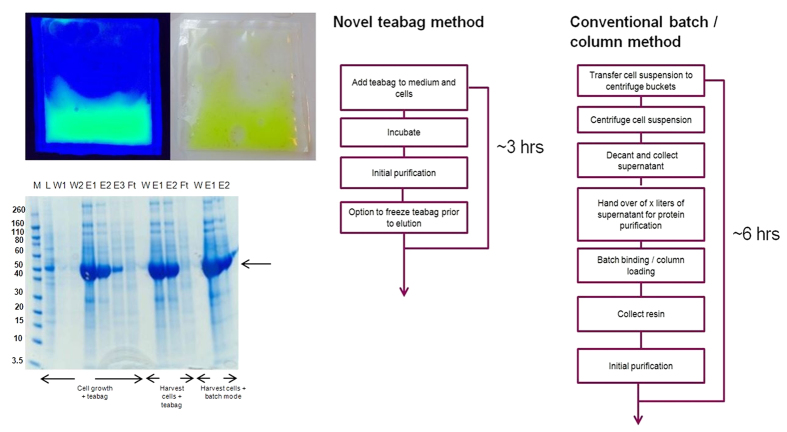
(**A**) A teabag containing Ni Sepharose Excel™ resin (GE Healthcare) and immobilized His-tagged GFP protein. The teabag is imaged under both UV and ambient light. (**B**) SDS-PAGE analysis of His-tagged mPAI1 expressed in CHO-EBNA GS cells. The gel shows the comparison between the batch method and the teabag method in different conditions. In all experiments 1 ml of Ni Sepharose Excel™ was used. The image shows that purification with the teabag method is comparable to conventional batch method. As shown there is almost nothing in the flow through and negliable amounts of protein in the wash fractions. M = Marker (Novex Sharp), L = Load media, Ft = Flow through fractions, W = Wash fractions, E = Elution fractions. (**C**) Schematic process overview and predicted time using the teabag method or conventional methods.

We have identified a low protein binding mesh, which can be heat-sealed to generate bags to contain affinity resins, similar to tea leaves confined in a teabag (Supplementary Fig. S1). Various mesh sizes were evaluated, and the 40 μm Sefar Petex™ mesh was chosen as it provided optimal physical characteristics: fluid exchange, confinement of a variety of affinity resins and exclusion of particulate matter. An example of an IMAC resin containing teabag is shown in Fig. 1A, His-tagged GFP protein is immobilized on the resin and imaged under UV and ambient light.

Figure 1B shows a direct comparison of three different purification schemes of secreted mPAI1 protein using a CHO-EBNA GS expression system^1^: teabag purification of protein from fermentation broth in the presence of cells and cell debris; teabags using clarified fermentation broth; and conventional batch purification on clarified fermentation broth. This secreted protein is captured completely in all three purifications, and yields and purity were comparable (~20 mg/L and 80%, respectively). Similar results were obtained using a HEK cell expression system (Supplementary Fig. S2; all experimental results are summarized in detail in Supplementary Table 1). Teabag purification is not limited to Ni-IMAC affinity resins only, Supplementary Fig. S3 shows effective capturing of VEGFR2-Fc using MabSelect SuRe™ affinity filled teabags.These results demonstrate that teabag purification is an excellent method for capturing proteins directly from the fermentation broth in the presence of cells, particulates and debris.

Next we established that teabag purification is a suitable method to recover low expressing proteins secreted in the medium. TGF1*β* has a expression level of approximately 1 mg/L and was efficiently purified from CHO- EBNA GS supernatant (Supplementary Table 1). Spiking experiments using His-tagged GFP demonstrated recovery from as little as 0.1 mg/L protein from mammalian cell medium. We further established that the Ni Sepharose Excel™ resin containing teabags do not significantly affect CHO cell growth for extended time periods, after 15 days viability was still above 97% with only a marignal viability decrease (<1%) compared to day 13. However, minor leakage of resin had occured compromising cell number measurements. Therefore, it is recommended to apply resin filled teabags after the final cell-count. Nevertheless, with further optimization of mesh and resin, combining growth and capturing is an attractive option, and with minor adaptations to growth vessels, allows seamless incorporation of teabag purification during mammalian cell growth.

We have demonstrated that teabag purification is an efficient method for purification of secreted proteins without prior removal of cells and debris, thus reducing total processing and hands on time considerably (Fig. 1C). However, teabag purification is also a suitable method for capturing intracellular proteins from lysates. Supplementary Table T1 gives a range of examples where we have applied teabag purification for non-secreted proteins produced in insect cell expression systems (Supplementary Fig. S4) and in *E. coli* based protein expression (Supplementary Fig. S5). Direct comparison of batch and teabag purification demonstrates equal capturing efficiency and purification for both methods. Moreover, we demonstrate that teabags can capture proteins without prior clarification of lysates illustrating that our teabag purification can reduce processing time also for intracellularly expressed proteins (Supplementary Fig. S6).

From a logistical point of view teabag purification has advantages as well, particularly in a global industrial setting were protein expression and purification may not always be co-localised. Here, instead of handling and transporting bulk medium, a number of teabags with affinity captured protein can be transported. Supplementary Figure S7 shows results of an experiment where a protein was captured using IMAC in a teabag and subsequently frozen. The teabag was frozen for over a week simulating transport, thereafter thawed and eluted. To imitate inadvertent thawing during transport, a set of teabags was frozen and thawed twice. The quality of the material, as judged by SDS-PAGE and size exclusion chromatography, was indistinguishable from protein material which had not been frozen. Obviously, this may depend on the specific protein target; nevertheless this shows that protein teabags can be frozen without detrimenal effects on elution properties and quality.

Expanded bed adsorption (EBA) purifications^2^ enables protein recovery from feedstock that contains cells, debris and particles. However, EBA requires specific adsorption columns and custom density graded affinity resin; furthermore EBA purification is less applicable for multiple parallel purifications. The teabag method presented here can be used with common laboratory affinity resins and the ease of handling supports parallel purification. The method is also highly scaleable by either increasing the size or the number of teabags. Circular teabags with matching diameters can be placed in to a column and subsequently treated similarly to ordinary column purifications. Thus, the teabag method is also applicable in cases where a gradient elution is required.

Porath and Sandberg described in 1971 a similar strategy for purification^3^, however the method was not widely adopted. One reason why our teabag purification method will be more widely applicable has been the development of novel affinity resins; particularly Ni-IMAC resins which are more resistant to chelating agents in media or lysates^4^. Purification with traditional IMAC resins have been limited due to the leakage of nickel ions, and hence reduced binding capacity.

We have developed a novel purification method, which facilitates capture and purification of proteins directly from the growth medium or lysate without requirements of centrifugation steps for clarification. This method is versatile and widely applicable, reduces handling time considerably and increases throughput without significant investments. Moreover the method can replace batch binding protocols.

## Materials and Methods

### Electrophoresis

Samples were denatured in LDS buffer, separated on 4–12% Bis-Tris NuPage gels (Thermo Scientific) and either stained with e-Stain device from GenScript or Instant Blue (Expedeon Ltd.)

### Choice of mesh

The choice of mesh was predominantly guided by heat sealing properties, while the pore-size was optimized to retain affinity resin while supporting fluid exchange. Sefar Nitex™ (Bigman AB, Sweden) and Sefar Petex™ (Bigman AB, Sweden) were evaluated; handling of the polyester based Petex™ mesh was most effective. Three different pore sizes, 17, 25 and 40 μm, where evaluated for retention of resins, and the largest 40 μm mesh was selected for all further experiments. Protein binding to Petex™ 40 μm mesh was quantified by BSA (Sigma A7906) recovery after incubation with mesh. The approximate area of a purification bag used in these studies is 50 cm^2^, less than 30 μg BSA was absorbed to this mesh area.

### Construction of teabags

Teabags were constructed from the nylon mesh using 6 × 12 cm of mesh, folding the mesh in the middle and heat sealing (Quick-Seal, O. Möllerström AB, 10 sec) along two edges to form a bag with final dimension of 4 × 4 cm after trimming the outer edges of the container. The 4 × 4 cm bags are optimized for 1–2 ml of resin, smaller bags 1.5 × 3 cm have been used for 0.3 ml of resin and for 5 ml resin we constructed 5 × 10 cm bags. The 4 × 4 cm teabags were filled with 1 ml resin of various affinity resins (*i.e*. MabSelect SuRe™, Ni Sepharose FF, Ni Sepharose Excel™ and cOmplete™ His-Tag Purification Resin) and then heat sealed along the last edge. Care is taken to avoid contact of the sealing area with resin as this could compromise the integrity of the seal. After filling and sealing of the teabag, the bags were washed with water and equilibrated with purification buffer prior to use. The resin filled bags can be stored in 20% ethanol. Non-filled bags can be sterilised by autoclaving at 121 °C using a standard program for dry equipment/utensils. After filling and sealing the sterile teabags with the chosen sterile resin, the teabags can be stored in 20% ethanol. Sterilised bags have been used in mammalian cultures for 15 days without compromising cell growth. Teabag purification has been applied to endotoxin free purification without further treatment of the mesh, but pre-made teabags can also be pre-treated with 0.1 M sodium hydroxide. A number of different shapes were evaluated: flat rectangular shapes, flat semi-circular shapes, tetrahedral-like, pyramids and tubes. Rectangular shapes were selected due to the ease of construction and since these supported elution of protein from the resin in small volumes better than the 3D shaped tetrahedral and pyramids.

### Expression of proteins using mammalian cells

Protein expression were performed in two established mammalian cell systems, Chinese hamster ovary cell line (CHO-EBNA GS)^5^ cells and human embryonic kidney cells (HEK293 6E, Life Technologies). CHO-EBNA GS cell expression was as described by Abbott *et al*.^1^. Briefly, CHO-EBNA GS cells were seeded at 0.5 × 10^6^  cells/ml in unsupplemented media (CD-CHO, Gibco) a day before transfection. At a cell density of 1–1.2 × 10^6^  cells/ml, cells were transfected with 0.5 mg/ml DNA and PEI MAX to a final concentration of 7 μg/ml. 24 h after transfection, the cells were fed with appropriate feed solution and on day 4 post-transfection, the temperature was shifted to from 37 °C to 30 °C. Cultures were harvest 14–15 days post-transfection, ensuring cell viability was over 90%. For HEK293 6E expression, cells were seeded at 0.8 × 10^6^ cells/ml and cultured for 24 h in F17 media (Invitrogen). At a cell density of 1.6–1.8 × 10^6^ cells/ml cells were transfected with 0.75 mg/ml DNA and PEI MAX to a final concentration of 2.8 μg/ml. 24 h after transfection, the cells were fed with HyPep 1510 (Kerry Biosciences) to a final concentration of 0.32% (v/v). Cultures were harvested before the cell viability dropped below 75% (6–8 days post-transfection). In cases of harvesting clarified media, cells and debris were removed by centrifugation at 3400 g for 15 min at room temperature. In cases where teabag purification was conducted at the end of cell fermentation, resin filled bags were added 2 hrs prior to termination and subsequently removed from the culture medium for washing and elution. In experiments where teabag purification is combined during cell growth, teabags were introduced aseptically to the cultures on day 1 of the growth and removed from the cultures on the day of harvest. Growth proceeded otherwise as indicated above.

### Teabag purification and freezing

A teabag was added to secreted protein media or lysate, incubated for 2 h or overnight. The teabag was transferred to a 50 ml falcon tube, washed with wash buffer (20 CV) and wash buffer with 50 mM imidazole (5 CV) followed by 7 × 1 CV elutions with 500 mM imidazole in buffer (see Supplementary Table 1 for all buffer details). While buffer systems typically may need optimization for individual proteins, we recommend PBS as initial starting buffer for teabag purification for secreted proteins. For intracellularly expressed proteins, a recommended starting buffer would contain 50 mM Tris/HCl, pH 8, 500 mM NaCl, and 1 mM TCEP.

In order to test freezability of protein immobilized on teabags, we have used an *E. coli* expressed human EF-hand domain, see also Supplementary Table 1. Cleared lysate was split into six 50 ml fractions, protein was captured using Ni-IMAC teabags as described and washed using two wash steps. After washing, two teabags were eluted as usual, while the four remaining teabags were transferred to plastic bags and frozen at −20 °C. After a week the teabags were thawed in room temperature, two teabags were eluted as usual, while the two remaining teabags were re-frozen at −20 °C for half an hour and thawed a second time. All eluates were flash frozen immediately after elution and thawed in water prior to size exclusion chromatography. The quality of the eluted protein was assessed using SDS-PAGE analysis and size exclusion chromatography using a BioRAD Enrich 70 column in 40 mM HEPES pH 7.4, 300 mM NaCl at 1.5 ml/min.

## Additional Information

**How to cite this article**: Castaldo, M. *et al*. A fast and easy strategy for protein purification using “teabags”. *Sci. Rep*. **6**, 28887; doi: 10.1038/srep28887 (2016).

## Supplementary Material

Supplementary Information
