# Supplementary material for: A fast and easy strategy for protein purification using “teabags”
Source: Sci Rep. 2016 Jun 30;6:28887. doi: 10.1038/srep28887 (PMC4928122; doi:10.1038/srep28887)
Supplement: Supplementary Information [file srep28887-s1.pdf]

Supplementary material accompanying “A fast and easy strategy for protein purification using ‘teabags’ ” by M. Castaldo, L. Barlind, F. Mauritzson, P. T. Wan, H.J. Snijder.

Supplementary Figure S1

A step-by-step illustration of the construction of a protein purification teabag.From left-to-right and from top-to-bottom Step 1: Cut a 6 x12 cm piece of mesh with clean sciccors. Step 2: Fold the mesh in the middle. Step 3 Heatseal along two of the open edges to yield a bag. Step 4: Load the bag with resin, taking care to apply the resin to the bottom of the bag. Step 5: Close the bag by heatsealing along the remaining open edge. The last image shows a filled teabag with His-tagged GFP immobilized on IMAC resin.

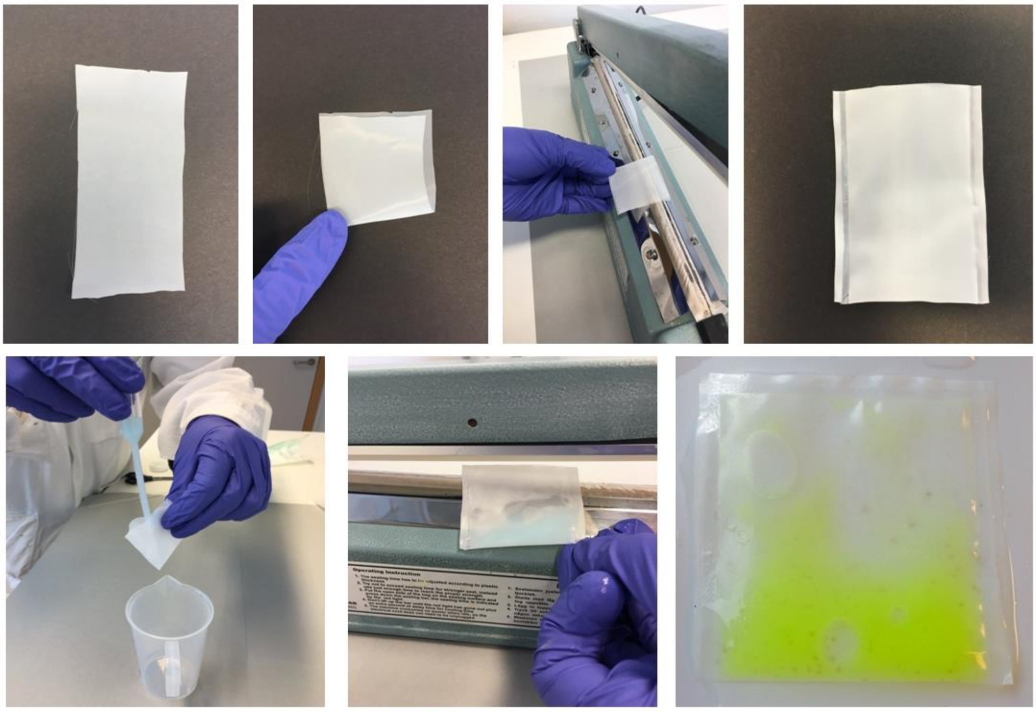

Supplementary Figure S1

Supplementary Figure S2

SDS-PAGE analysis of his-tagged mPAI expressed in HEK cells, the 48 kDa mPAI is indicated by an arrow. The gel shows the comparison between the teabag purification and batch method. In all experiments 1 ml of Ni Excel™ (GE Healthcare) was used. The image shows that purification with the teabag method is comparable with conventional batch method. As shown there is almost no protein in the flow through and neglectable amounts of protein in the wash fractions. M = Marker (Novex Sharp), L = Load media, Ft = Flow through fractions, W = Wash fractions, E = Elution fractions

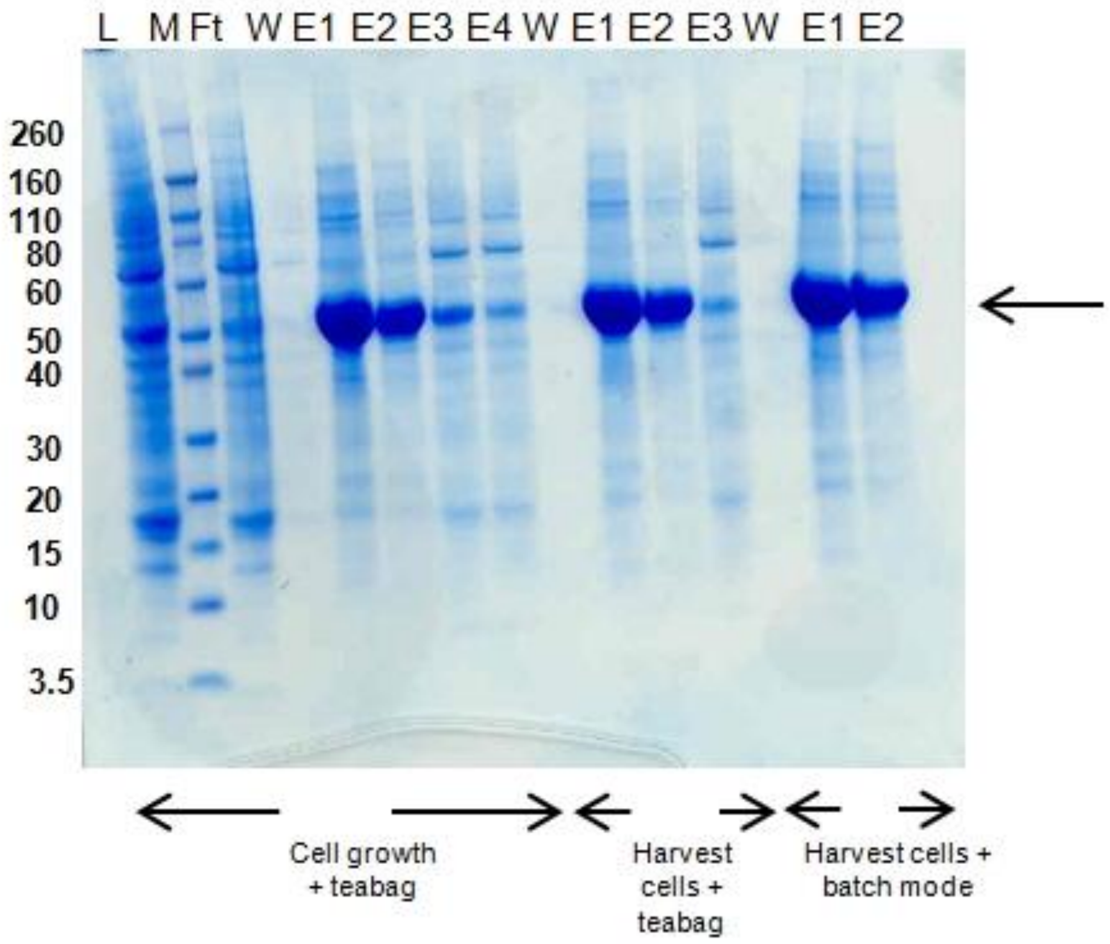

Supplementary Figure S2

Supplementary Figure S3

Comparison of conventional packed column purification *vs.* teabag purifications of Fc-tagged VEGFR2 receptor using MabSelect SuRe™ affinity resin (GE Healthcare), the 110.8 kDa protein dimer is indicated by an arrow. The volume of resin in the two experiments was 1 ml. In these experiments the binding capacity of the resin was exceeded, as is apparent from the excess material in the non-bound fraction. A duplicate teabag purification showed nearly identical results (results not shown). M = Marker, L = Load media, Ft = Flow through fractions, W = Wash fractions, E = Elution fractions. For the teabag purification two dilutions have been loaded on gel, the latter corresponds to an equal amount as for the column purification.

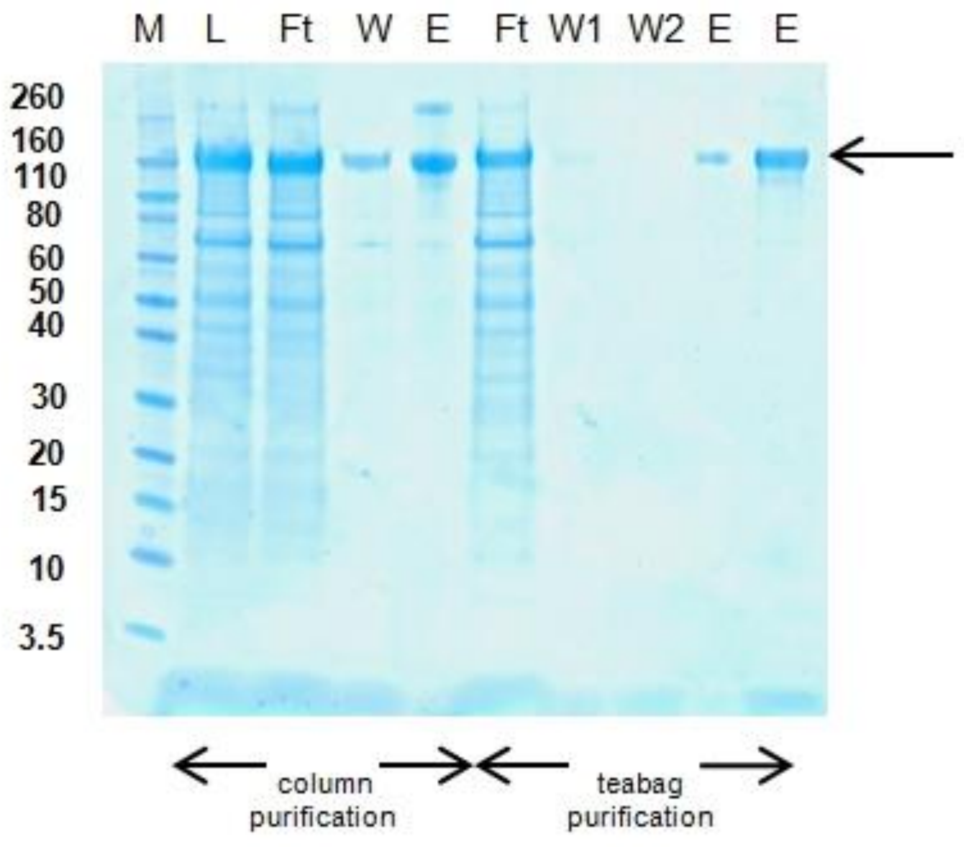

Supplementary Figure S3

Supplementary Figure S4

SDS-PAGE analysis of a full length His-tagged intracellular protein expressed in *Sf21* insect cells, the 142 kDa protein is indicated by an arrow. Figure S4 shows the purification using the teabag method. Ni Sepharose (GE Healthcare) was used. M = Marker, L = Clarified lysate, Ft = Flow through fractions, W = Wash fractions, E = Elution fractions.

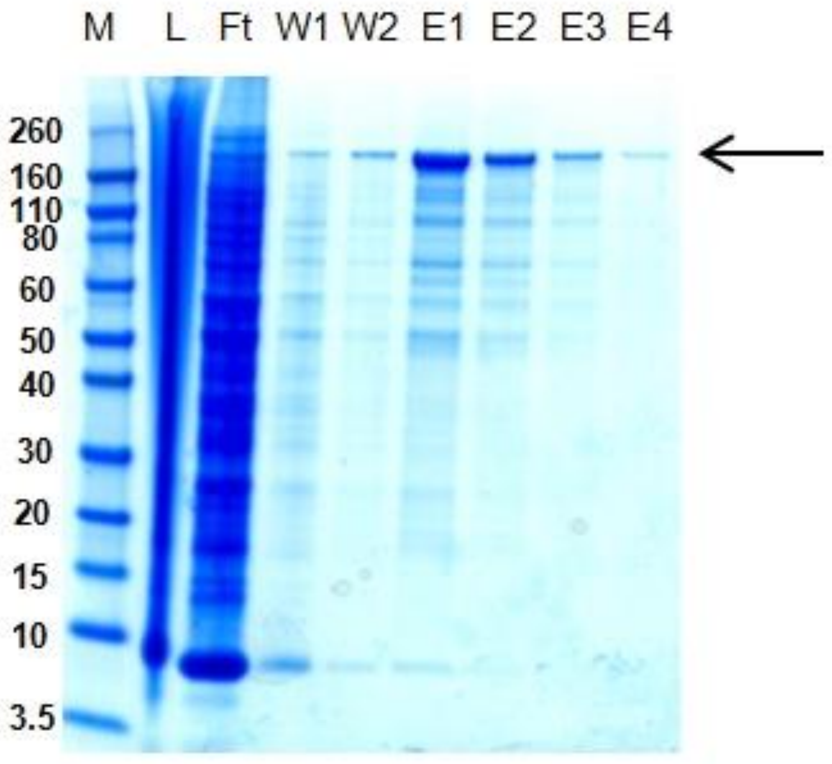

Supplementary Figure S4

Supplementary Figure S5

SDS-PAGE analysis of His-tagged Keap-1 expressed in *E. coli*, the 34 kDa protein is indicated by an arrow. Supplementary Figure S5 shows the comparison between the teabag method (left) and the batch method (right). In the comparison 1 ml of Ni Sepharose FF (GE Healthcare) was used. The images show that purification with the teabag method is equal to or better than the conventional batch method. As shown there is almost no protein in the flow through and negligable amounts of protein in the wash fractions. M = Marker, L = Clarified lysate, Ft = Flow through fractions, W = Wash fractions, E = Elution fractions.

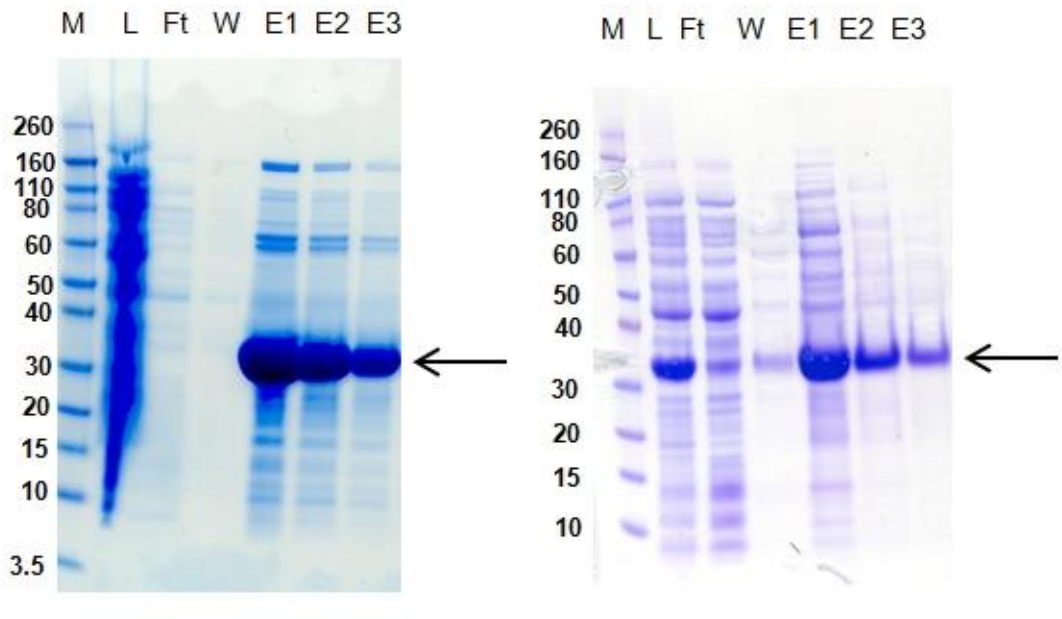

Supplementary Figure S5

Supplementary Figure S6

SDS-PAGE analysis of a 13.0 kDa human EF-hand protein domain (indicated by an arrow) expressed intracelllularly in *E. coli*. Supplementary Figure S6 shows a gel comparing IMAC affinity capture of the protein form cleared lysate or total non-clarified lysate using the teabag method. Ni Sepharose FF (GE Healthcare) was used as affinity resin. Each sample was loaded in duplicates. M = Marker, L = Lysate, Ft = Flow through fractions, W = Wash fractions, E = Elution fractions, tot = total lysate, clr = cleared lysate.

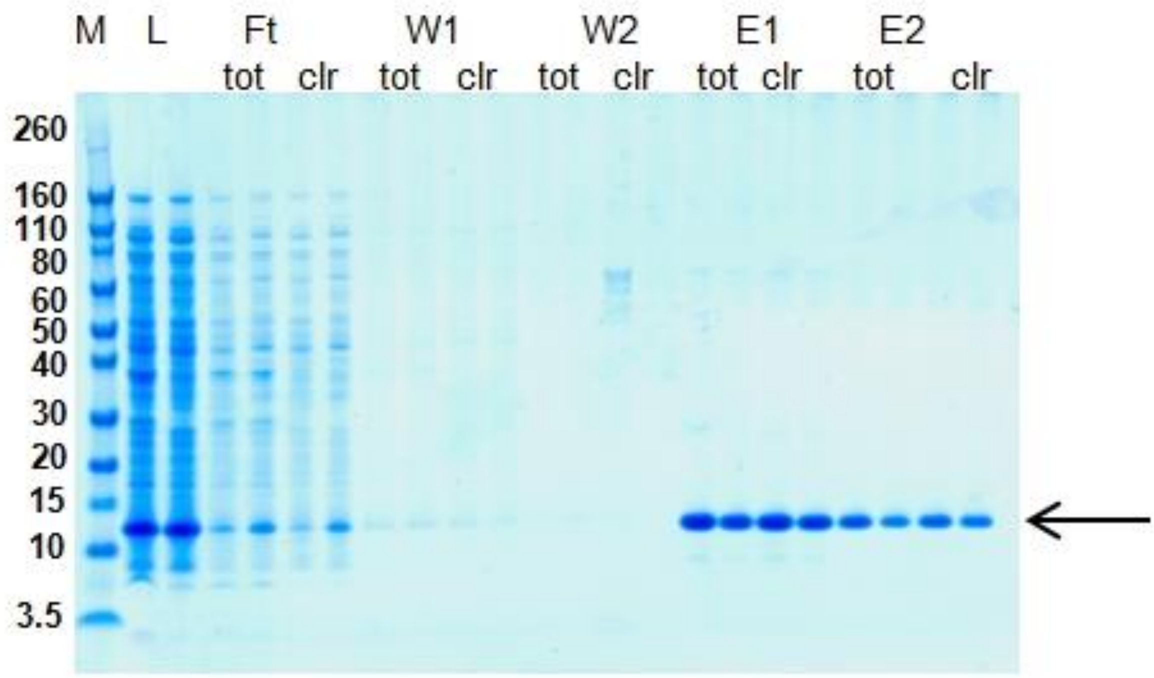

Supplementary Figure S6

Supplementary Figure S7

Supplementary Figure S7A shows representative size exclusion chromatogaphic traces of a 13.0 kDa EF-hand protein domain purified using IMAC teabags from total *E. coli* lysate or after lysate clarification, and after freezing the teabag with the affinity captured protein once or twice. The teabags were kept frozen for one week prior to thawing, elution and subsequent size exclusion chromatographic separation. Supplementary Figure S7B shows the integrated peak intensities from duplicate experiments. The figures illustrate that this protein immobilized on resin inside teabags can be frozen without significant effect on the protein quality as judged by SEC.

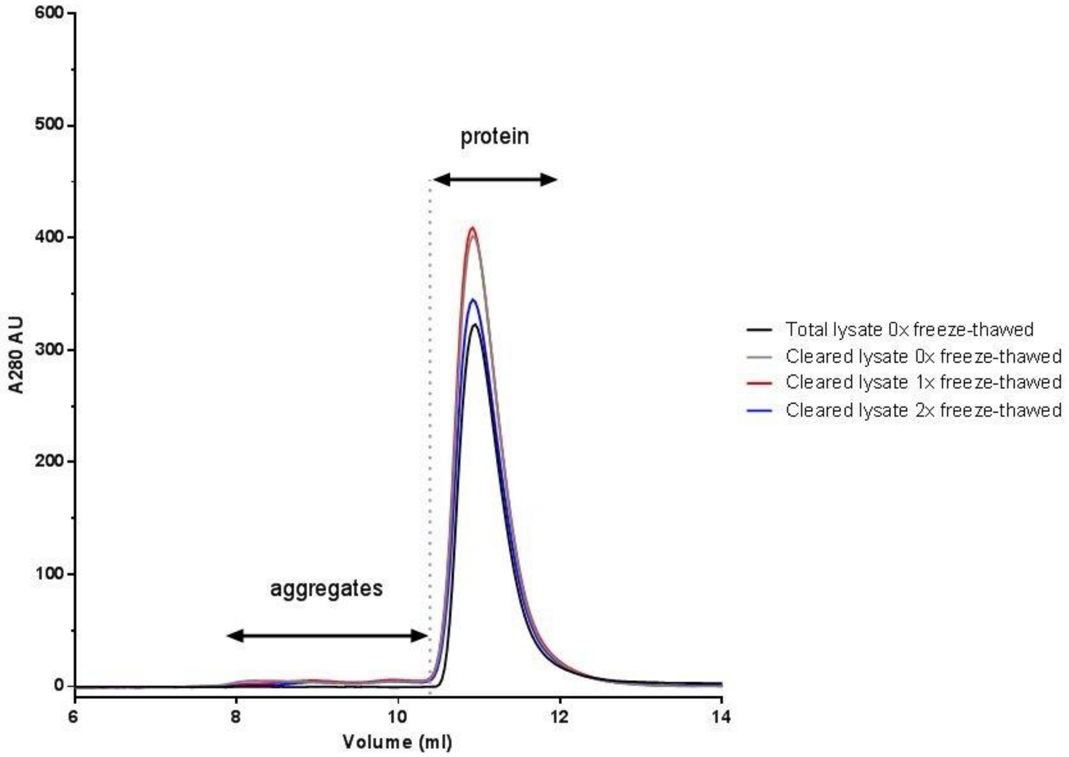

Supplementary Figure S7 A

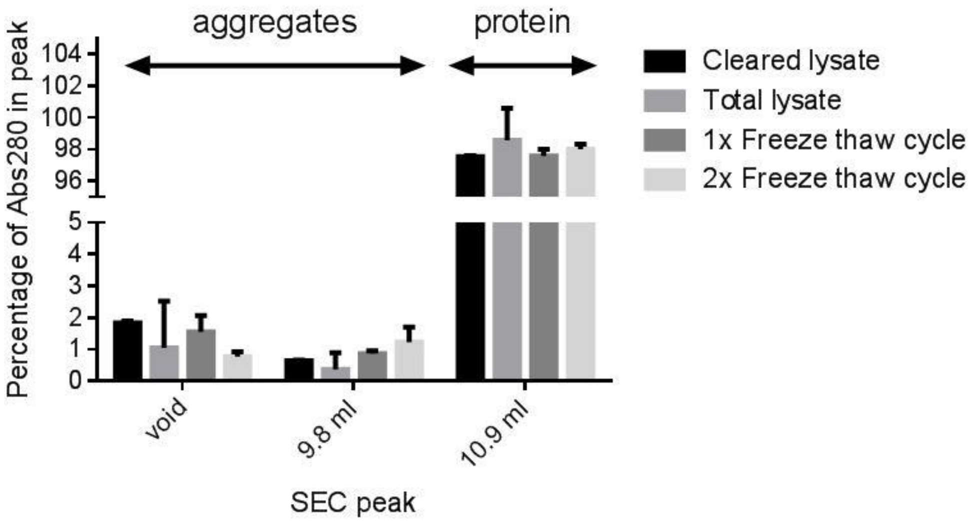

Supplementary Figure S7 B

Supplementary Table 1 accompanying “A fast and easy strategy for protein purification using ‘teabags’ ” by M. Castaldo, L. Barlind, F. Mauritzson, P. T. Wan, H.J. Snijder.

Summary of different protein targets and expression systems where conventional purification methods have been compared with the teabag method. In some cases the teabag method has been used exclusively.

**Target**	**expression system**	**culture volume (ml)**	**resin**	**resin volume (ml)**	**method**	**wash buffer**	**elution buffer**	**elution (ml)**	**purity (%)**	**yield (mg)**	**yield (mg/L)**
**mPAI**	CHO-EBNA GS	100	Ni Sepharose Excel	1	batch	1xPBS	1xPBS, 500 mM Imidazole	7x1 ml	50	2	20
**mPAI**	CHO-EBNA GS	100	Ni Sepharose Excel	1	batch cleared growth media	1xPBS	1xPBS, 500 mM Imidazole	7x1 ml	50	2	20
**mPAI**	CHO-EBNA GS	100	Ni Sepharose Excel	1	teabag	1xPBS	1xPBS, 500 mM Imidazole	7x1 ml	50	2	20
**mPAI**	HEK293 6E	100	Ni Sepharose Excel	1	batch	1xPBS	1xPBS, 500 mM Imidazole	7x1 ml	50	1.7	17
**mPAI**	HEK293 6E	100	Ni Sepharose Excel	1	batch cleared growth media	1xPBS	1xPBS, 500 mM Imidazole	7x1 ml	50	1.7	17
**mPAI**	HEK293 6E	100	Ni Sepharose Excel	1	teabag	1xPBS	1xPBS, 500 mM Imidazole	7x1 ml	50	1.7	17
**TGFb-1**	CHO-EBNA GS	3000	Ni Sepharose Excel	5	teabag	1xPBS	1xPBS, 500 mM Imidazole	7x5 ml	>95	3	1

**VEGFR2-Fc**	CHO-EBNA GS	70	MabSelect SuRe	1	column	1xPBS	50 mM Glycine pH 3.0, 0.1 M NaCl	4 ml	> 95	4	57
**VEGFR2-Fc**	CHO-EBNA GS	5	MabSelect SuRe	1	teabag	1xPBS	50 mM Glycine pH 3.0, 0.1 M NaCl	1 ml	> 95	0.3	60
**scFv**	CHO-EBNA GS	250	Ni Sepharose FF	2	batch	25 mM Tris/HCl, pH 7.5, 300 mM NaCl, 20 mM Imidazole, 1 mM TCEP	25 mM Tris/HCl, pH 7.5, 300 mM NaCl, 500 mM Imidazole, 1 mM TCEP	5x2 ml	<60	2.1	8.4
**scFv**	CHO-EBNA GS	250	Ni Sepharose Excel	1	batch	25 mM Tris/HCl, pH 7.5, 300 mM NaCl, 20 mM Imidazole, 1 mM TCEP	25 mM Tris/HCl, pH 7.5, 300 mM NaCl, 500 mM Imidazole, 1 mM TCEP	5x1 ml	30	2	8
**scFv**	CHO-EBNA GS	250	Ni Sepharose FF	1	teabag	25 mM Tris/HCl, pH 7.5, 300 mM NaCl, 20 mM Imidazole, 1 mM TCEP	25 mM Tris/HCl, pH 7.5, 300 mM NaCl, 500 mM Imidazole, 1 mM TCEP	7x1 ml	>60	4.4	17.6
**intracellular prot**	*Sf21*	500	Ni Sepharose FF	1	batch	50 mM Tris/HCl, pH 8, 500 mM NaCl, 200 mM Arginine, 1 mM TCEP, 0.2 mM Na orthovanadate, 0.25 mM Na pyrophosphate, 0.5 mM NaF	50 mM Tris/HCl, pH 8, 500 mM NaCl, 200 mM Arginine, 1 mM TCEP, 500 mM Imidazole, 0.2 mM Na orthovanadate, 0.25 mM Na pyrophosphate, 0.5 mM NaF	7x1 ml	55	4	8
**intracellular prot**	*Sf21*	500	Ni Sepharose FF	1	teabag	50 mM Tris/HCl, pH 8, 500 mM NaCl, 200 mM Arginine, 1 mM TCEP, 0.2 mM Na orthovanadate, 0.25 mM Na pyrophosphate, 0.5 mM NaF	50 mM Tris/HCl, pH 8, 500 mM NaCl, 200 mM Arginine, 1 mM TCEP, 500 mM Imidazole, 0.2 mM Na orthovanadate, 0.25 mM Na pyrophosphate, 0.5 mM NaF	7x1 ml	55	4.5	9
**Keap-1**	*E.coli*	300	Ni Sepharose FF	1	batch	50 mM Tris/HCl, pH 8, 500 mM NaCl, 20 mM Imidazole, 10 % glycerol, 1 mM TCEP	50 mM Tris/HCl, pH 8, 500 mM NaCl, 300 mM Imidazole, 10 % glycerol, 1 mM TCEP	10x1 ml	>60	8.4	28

**Keap-1**	*E.coli*	300	Ni Sepharose FF	1	teabag	50 mM Tris/HCl, pH 8, 500 mM NaCl, 20 mM Imidazole, 10 % glycerol, 1 mM TCEP	50 mM Tris/HCl, pH 8, 500 mM NaCl, 300 mM Imidazole, 10 % glycerol, 1 mM TCEP	10x1 ml	>60	9	30
**Rat flavin containing enzyme 1**	*E. coli*	375	Ni-NTA Super Flow	1	batch	50 mM HEPES pH 7.3, 100 mM NaCl, 10% glycerol and 1 mM TCEP	50 mM HEPES pH 7.3, 100 mM NaCl, 10% glycerol 300 mM Imidazole and 1 mM TCEP	5 ml	75%	4.5	12
**Rat flavin containing enzyme 1**	*E. coli*	375	Ni-NTA Super Flow	1	teabag	50 mM HEPES pH 7.3, 100 mM NaCl, 10% glycerol and 1 mM TCEP	50 mM HEPES pH 7.3, 100 mM NaCl, 10% glycerol 300 mM Imidazole and 1 mM TCEP	5 ml	75%	6.5	17.3
**Rat flavin containing enzyme 2**	*E. coli*	375	Ni-NTA Super Flow	1	teabag	50 mM HEPES pH 7.3, 100 mM NaCl, 10% glycerol and 1 mM TCEP	50 mM HEPES pH 7.3, 100 mM NaCl, 10% glycerol 300 mM Imidazole and 1 mM TCEP	5 ml	75%	5	13.3
**Human EF-hand domain protein**	*E. coli*	175	Ni-NTA Super Flow	1	teabag cleared lysate	40 mM HEPES pH 7.4, 300 mM NaCl	40 mM HEPES pH 7.4, 300 mM NaCl, 300 mM Imidazole	5 ml	95%	6.5	37.1
**Human EF-hand domain protein**	*E. coli*	175	Ni-NTA Super Flow	1	teabag non-cleared lysate	40 mM HEPES pH 7.4, 300 mM NaCl	40 mM HEPES pH 7.4, 300 mM NaCl, 300 mM Imidazole	5 ml	95%	6.3	36
